# A Randomised Controlled Trial of Early Vitrectomy and Intravitreal Antibiotics for Post-operative Exogenous Endophthalmitis (EVIAN study): study protocol for a feasibility trial

**DOI:** 10.3310/nihropenres.13469.1

**Published:** 2023-09-28

**Authors:** Mahiul Muhammed Khan Muqit, Carlos Pavesio, Hayley Boston, Keerththika Sriharan, Yanzhong Wang, Elena Pizzo, Stephen Cobb, Christopher Spink, James Bainbridge

**Affiliations:** 1 Vitreoretinal Service, Moorfields Eye Hospital, London, UK; 2 Institute of Ophthalmology, University College London, London, England, UK; 3 Uveitis Service, Moorfields Eye Hospital, London, UK; 4 Research and Development, Moorfields Eye Hospital, London, UK; 5 School of Life Course and Populational Sciences, Kings College London, London, UK; 6 Department of Applied Health Research, University College London, London, England, UK; 7 The Royal Courts of Justice, London, UK; 8 Patient Representative, Moorfields Eye Hospital, London, UK

**Keywords:** Endophthalmitis; vitrectomy, intravitreal antibiotics; randomised controlled trial; visual acuity; complications

## Abstract

Postoperative bacterial endophthalmitis is an infection of the eye's internal tissues resulting from an intraocular procedure. The condition is uncommon but can cause severe and irreversible impairment of sight. Standard management involves administration of antibiotics with or without subsequent removal of the infected vitreous gel by vitrectomy surgery. Surgical intervention is typically reserved for infections that persist despite a period of medical management alone. We aim to determine whether outcomes can be improved by performing surgery without delay. To explore the feasibility of a definitive randomised controlled trial and to determine the number of participants required, we will conduct a multicentre feasibility trial. This trial will include 40 affected individuals, allocated randomly to either standard of care, being intravitreal antibiotic administration alone, or to early vitrectomy surgery in addition to antibiotic administration. We will determine the feasibility and size of a definitive trial by evaluating the participants and the outcomes for their sight. Research Ethics Committee approval (REC 20/WM/0264). Here we describe the trial protocol.

**Trial registration number:**ClinicalTrials.gov NCT04522661

## Plain English summary

This study aims to assess the feasibility of a trial to determine the benefit of prompt surgery for “endophthalmitis”, which is a rare but blinding eye infection resulting from invasive eye procedures. Endophthalmitis can cause rapidly progressive, severe pain and irreversible loss of sight. Antibiotic administration alone may be insufficient to control the infection and in up to 50% of cases surgery is subsequently performed to improve the outcome by removing the infected vitreous gel (vitrectomy). Usual care involves intensive and extended inpatient and outpatient management over a 6-month period in specialist tertiary centres at considerable cost to the NHS.

Surgical intervention is typically reserved for infections that persist despite a period of medical management with intravitreal antibiotics alone. We aim to determine whether outcomes can be improved by performing surgery to remove infected vitreous gel without delay by removing infected vitreous material at the first opportunity. Early vitrectomy surgery has the potential to improve the outcome for people's sight and well-being, and to reduce the associated financial costs to the NHS, and assist in endeavours to reduce antibiotic usage.

To find out if earlier surgery can improve the outcome, we first need to determine if an appropriate clinical trial is feasible and decide how it should best be designed. Postoperative endophthalmitis is typically unexpected, causes considerable distress and places demand on urgent clinical services. We need to confirm that a sufficient number of affected people would be willing to participate in such a trial and that the hospitals providing care would have the capacity to perform early surgery.

Participants will be randomly allocated to vitrectomy performed at an earlier stage of the condition, or later if needs be in accordance with standard care. Standard follow-up for both arms will continue for a six-month period.

## Introduction

Endophthalmitis is a rare but potentially blinding eye complication that may occur following any invasive ocular procedure. Invasive ocular procedures are now very common, more so than ever before. Cataract surgery is the most common operation in the UK. Exudative/wet age-related macular degeneration (AMD) and diabetic macular oedema (DMO) are two leading causes of sight loss in the UK and these conditions may involve monthly eye injections into the vitreous jelly/humour "intravitreal injections." With the increasing numbers of invasive eye procedures, the frequency of postoperative endophthalmitis (POE) though rare is increasing ^1, 2^.

The Royal National Institute of Blind People (RNIB) ^3^ published a document in 2009 “Future Sight Loss: an epidemiological and economic model.” The RNIB state that although the severe surgical complication of endophthalmitis is are after cataract surgery, this complication impacts patients’ quality of life and result in vision loss despite treatment ^3^. At the time, UK endophthalmitis incidence was 0.51 per 1000 operated eyes. The 2009 report estimated that in 2010, for 199 cases of endophthalmitis, the financial disease burden for cataract surgery would be £995,144,453. With a higher incidence in 2010 (around 1.3 per 1000 operated eyes) this would result in 510 cases, at a total cost of illness of £996,323,311. These additional 311 additional cases would create extra costs of about £1,178,859 million. Consequently, the rising incidence of endophthalmitis in 2010 will lead to 160 people with uni-ocular sight loss. The estimated number of cases of endophthalmitis following cataract surgery in 2020 is estimated to increase to 203 cases (England), 13 cases (Wales), 20 cases (Scotland) that gives an estimated increased total of 236 cases per 473,944 cataract surgeries ^3^. The RNIB report that although endophthalmitis is a very rare event, it is alarming that a significant percentage of patients will suffer serious loss of sight or loss of the eye, and the condition often difficult to treat and can be very costly to manage.

The seminal study into the role of vitrectomy for POE following cataract surgery is the Endophthalmitis Vitrectomy Study (EVS) published in 1995 ^4^. The findings suggested that surgery to remove infected vitreous by pars plana vitrectomy (PPV) offered an improved outcome for individuals with vision limited to perception of light (POL) or poorer. The EVS found 33% of patients who underwent PPV with POL vision had visual acuity (VA) 20/40 or better at final follow up, and 56% of patients to achieve VA better than 20/100.

EVS assessed endophthalmitis treatment only in post-cataract surgery patients. No RCT evidence to answer the question of early vitrectomy surgery for the treatment of endophthalmitis following any eye surgery (retinal, glaucoma, corneal). Given the new treatment paradigms for wet AMD and DMO, much more frequently performed ocular injections have been identified to date. In the last 23 years, the standard clinical practice for all types of POE that has developed following intra-ocular surgery has mostly followed guidance of the EVS in the absence of any new randomised controlled trial (RCT). In the real world, this timely implementation of an immediate vitrectomy surgery is variable based on availability of theatre resources, so can be delayed for several days to weeks. In the last 10 years, vitrectomy surgery has evolved significantly with the introduction of small gauge PPV (23-, 25-, 27-gauge), wide-angle viewing systems and the increased routine use of silicone oil: none of which were used in the EVS study.

The prevalence of endophthalmitis in the UK is not publicly available but it is well recognized that endophthalmitis presents a significant risk of irreversible blindness. At Moorfields Eye Hospital, we publish annual prevalence rates and compare these against Royal College of Ophthalmology benchmarks. The Moorfields 2018–2019 endophthalmitis incidence rates ranged from 0.17% (post-surgery) and 3.33% (post-injections). In a 5-year Moorfields study (2013–2018) ^5^, 35 patients underwent vitrectomy surgery for endophthalmitis; post-phacoemulsification (n=9); post-glaucoma surgery (n=5); post-intravitreal injection (n=7); post-corneal graft transplant (n=4); and, post-vitreoretinal surgery (n=3). 63% patients had baseline visual acuity (VA) of perception of light vision. At average follow-up of 17 months, 11% had VA of 6/12 or better, and 26% had VA of 6/36 or better. Vitrectomy within 5 days of presentation resulted in significantly greater mean LogMAR VA gain of 1.12 compared to 0.6 following vitrectomy after 5 days (p= 0.011) demonstrating a benefit of earlier vitrectomy surgery ^5^.

A 2005 study where 23-gauge, early PPV was performed on 47 patients with VA better than POL reported 91% of patients achieving final VA of 20/40 ^6^. The author did not report pre-operative VA and there was no specific VA threshold prior to PPV. Other recent studies have demonstrated 75% achieving VA better than 20/40, and 80% better than 20/50 ^7, 8^. These outcomes compare favourably to the 53% having VA better than 20/40 in the EVS. In glaucoma, bleb-associated POE has a poor visual outcome, and PPV surgery in these cases has been shown to produce more favourable outcomes for patients ^9^. These reported findings and a better understanding of the pathology of endophthalmitis have led clinicians to re-consider the role of early PPV in POE.

The standard of care for endophthalmitis is an immediate vitreous gel biopsy with injection of two intravitreal antibiotics. The response and vision are assessed at 24 to 48-hours. If positive, then a further 48-hour period of observation follows. If the patient’s condition worsens, further antibiotic injections are given. However, if the deterioration is severe with VA reduced to POL, only then will patients be assessed by vitreoretinal surgeons for consideration of vitrectomy.

The authors (MM, JB) conducted a review to assess the potential role of combined PPV and intravitreal antibiotics in the acute management of exogenous endophthalmitis, versus the standard of care, defined as vitreous tap and intravitreal antibiotics ^10, 11^. Only a single RCT ^4 ^was identified for the role of early vitrectomy in exogenous endophthalmitis. Our data synthesis suggested that there may be no difference between groups (VIT vs TAP) for VA at three- or nine-months’ follow-up.

There is a clear need for reliable evidence to determine the benefit of early vitrectomy surgery in postoperative endophthalmitis. The Moorfields ocular inflammation/medical retina services, which oversee the endophthalmitis hospital protocols, have already advocated earlier vitrectomy to be performed at vision levels better than the EVS standards. Given the increasing demand for earlier intervention for endophthalmitis, and advancements in vitreoretinal surgery, there is a need for a new trial to investigate the role of contemporary vitrectomy management of acute endophthalmitis occurring as a complication of a larger spectrum of eye surgery or injection procedures.

Endophthalmitis patients spend often far more than 6 months attending hospital requiring intensive and sustained medication. Early vitrectomy surgery has the potential to reduce the overall treatment period, reduce the time spent by patients attending the hospital for clinical and surgery appointments, reduce the risk of severe visual loss, and ultimately accelerate visual recovery. The psychological impact is also significant with anxiety and depression a common feature. as well as considerable pain and vision loss. Societal costs could be reduced, including productivity losses for patients and their families.

The aim of early surgery to treat the endophthalmitis condition is to ultimately prevent severe visual loss. We hypothesise that performing early vitrectomy with intravitreal antibiotics compared to standard care repeat intravitreal antibiotics in the management of POE will accelerate recovery and improve visual outcomes. We predict that sufficient patients and surgeons would be willing to participate in a clinical trial to address the question. The present study aims to assess the feasibility of conducting a RCT comparing early vitrectomy surgery to the current standard patient care. If we can confirm that such a trial is feasible, we will plan a definitive randomized controlled trial evaluating its effectiveness. Here we describe the protocol for the feasibility trial.

## Methods

### Study design

**Figure 1.**
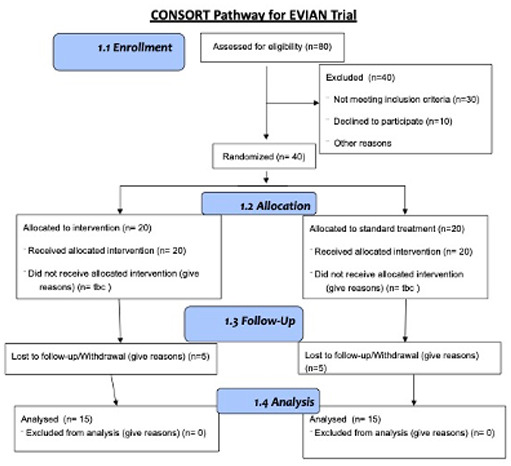
Study flow diagram for the EVIAN trial.

This is an open label, feasibility randomised controlled study of *E* arly *V* itrectomy and *I* ntravitreal *A* ntibiotics for Post-operative Exogenous E *N* dophthalmitis (EVIAN study) with an embedded health economic sub-study ( Figure 1).

### Setting

The trial will be conducted across at least 20 NHS ophthalmic centres in the United Kingdom. These centres manage patients with endophthalmitis with a specialist team of vitreoretinal surgeons, medical retina specialists, and uveitis specialists.

### Study participants

As part of standard NHS care, patients present at the eye clinic or emergency eye casualty and are diagnosed with POE. Potentially eligible participants with POE will be approached by the clinical team after their first presentation to the eye clinic. Adequate pain relief will be provided and nursing support available if further emotional support is required. All discussions take place in a private room away from the main clinical area. The patient will be informed of the study upon initial presentation with POE, and an EVIAN study Patient Information Sheet will be provided to the patient by the doctor. They will be given time to reflect, consider their decision with the opportunity to ask questions at that time or later. This first patient contact is when the initial diagnosis of POE is made, and the patient will immediately undergo standard of care vitreous biopsy (“tap”) and injection of intravitreal antibiotics.

***Eligibility***

Patients will be eligible for this study if they are:

- The patient over 18 years of age
- Patient has capacity to give informed consent
- Patient has not previously been enrolled in this study in regards to their other eye
- Diagnosis of postoperative endophthalmitis at any time-point following an ocular surgery/procedure/injection
- Symptomatic visual loss attributable to POE
- Best corrected visual acuity worse than 35 ETDRS letters, including counting fingers, hand motions and POL vision

We will exclude those:

- Patient suffered a major thromboembolic event within the past 3 months as (defined as transient ischaemic attack, Stroke, or myocardial infarction)
- Known adverse reaction to intravitreal antibiotics (amikacin/vancomyin/cephalosporins)
- Blood pressure greater than 200 systolic or 100 diastolic
- Any other condition that in the opinion of the investigator would preclude participation in the study (such as unstable medical status or severe disease that would make it difficult for the patient to be able to complete the study)
- The patient will use a research investigational drug during the study
- History of optic atrophy in the study eye
- Corneal oedema/haze that would prevent visualisation of fundus to perform vitrectomy surgery
- Patient over the age of 95 who present with POE should immediately be placed on the pre-screening log as ineligible

### Recruitment of participants, screening and eligibility assessment

The main sponsor site and all collaborating centres will engage with their infection control teams to set-up an alert of new index POE case (s) that can be communicated without delay to the site principal investigator and the research trial manager. The EVIAN study would be discussed internally at each site’s service meetings to promote awareness of the study to other colleagues (e.g. cataract, medical retina teams).

On diagnosis of a new POE case, the patient would be managed with an initial tap and inject POE visit. The patient will then be reviewed at 48 hours following this initial treatment. The study information provision is 48 hours, and this 48-hour additional time will allow participants enough time to have reflected on the study and make an informed decision. At the 48-hour visit, if the inflammatory signs, (hypopyon, red reflex) are improving with ETDRS vision better than 35 letters, then the patient is ineligible for trial, and patient will follow standard of care management outside the trial.

However, at the 48 hour visit, if the endophthalmitis has either not improved, or the situation is deteriorating with VA 35 ETDRS letters or worse, then the patient is eligible for the trial. The consent and randomisation will occur at this point. This enrolment/randomisation visit is designated day 0 for the trial.

### Informed consent, baseline assessment and trial specific screening tests

The participant will be instructed to return to the eye clinic after 48 hours from their initial assessment. At this point, the research medical team will review the participant’s clinical condition. If the participant now meets the study criteria, they will be invited to participate in the study. If they agree, they will be asked to sign the consent form. Patients either not fulfilling the trial inclusion criteria, or declining to participate in the study, they will be returned to standard NHS care.

Once the informed consent process is complete and the participant has reached a decision as to whether to participate, the investigator should record the decision in the case history form. A participant who decides to participate should be asked to sign the Informed Consent Form. A copy of the signed form should be given to the participant; one copy filed in medical records; and, the original kept in the Investigator Site File.

The baseline tests include history and clinical examination, blood pressure, ETDRS VA testing, ocular ultrasound, anterior segment photography, and quality of life questionnaires (detailed in section “Embedded health economic study” below). The patients will be followed up as shown in Table 1.

**Table 1.** Participant timeline.

Trial Weeks	Trial Visit Number	Early Vitrectomy Group Arm	Control Group Arm
-1 to 0		Records based Identification of potentially eligible treatment naive patients, assessment, issue of PIS, provisional recruitment, injections of intravitreal amikacin or ceftazidime, plus vancomycin. Vitreous biopsy taken Patient Information Sheet given to patient	
0 (48 hours post presentation of POE)	Baseline (Day 0)	**RETURN TO STANDARD CARE** **OR** **ENROLMENT** Fulfills eligibility and willing to participate Confirmation of eligibility, formal recruitment and randomization. Consent taken	
		Baseline Tests ETDRS VA testing Photography Ultrasound VFQ-25 EQ-5D CSRI	Baseline Tests ETDRS VA testing Photography Ultrasound VFQ-25 EQ-5D CSRI
0–48 hours (from Baseline)	1	Vitrectomy, vitreous biopsy plus intravitreal antibiotics (consent for surgery taken as for standard care)	Repeat intravitreal antibiotics
Day 2	2	Clinical Assessment in clinic ETDRS VA testing Ultrasound	Clinical Assessment in clinic ETDRS VA testing Ultrasound
Day 5–7	3	Clinical Assessment in clinic ETDRS VA testing	Clinical Assessment in clinic ETDRS VA testing
Day 8–14	4	Clinical Assessment in clinic ETDRS VA testing	Clinical Assessment in clinic ETDRS VA testing *Potential Vitrectomy surgery* *Day 12–15*
4 weeks	5	Clinical Assessment in clinic ETDRS VA testing Optical Coherence Tomography Fundus Photography	Clinical Assessment in clinic ETDRS VA testing Optical Coherence Tomography Fundus Photography
8 weeks	6	Clinical Assessment in clinic ETDRS VA testing Optical Coherence Tomography	Clinical Assessment in clinic ETDRS VA testing Optical Coherence Tomography
12 weeks	7	Clinical Assessment in clinic ETDRS VA testing Optical Coherence Tomography Optometric Refraction VFQ-25 EQ-5D CSRI	Clinical Assessment in clinic ETDRS VA testing Optical Coherence Tomography Optometric Refraction VFQ-25 EQ-5D CSRI

### Randomisation

Once the patient has completed enrolment, anonymous data will be entered into the online Castor database. The electronic Sealed Envelope Company platform will be used to randomise patients to groups at a 1:1 ratio to the Treatment or Control arm. Block size will be unknown to the investigators. Patients will undergo either vitrectomy within 48hours of randomisation; or, repeat intravitreal antibiotic injection 48 hours post-presentation that will be on the same day of randomisation. It is not possible to mask patients with regards to vitrectomy surgery, therefore it is reasonable to inform the patient if they are having an operation or not. Where two eyes of a participant meet inclusion criteria, the more affected eye in terms of BCVA will be selected for entry into the study. Where possible, patients will be informed immediately of their randomised group allocation in clinic.

### Interventions

***Vitrectomy Surgery-Treatment Arm***

Surgery will be performed by consultant vitreoretinal surgeon, or by a vitreoretinal fellow. The choice of anaesthesia is dependent on surgeon, patient choice and local standard practices. The PPV typically uses 3-port 27, 25 or 23-gauge sutureless ports. Twenty-gauge PPV can be used if this is the surgeon’s preference. The vitreous biopsy is taken before the infusion is started, and samples sent to the site’s local microbiology and micropathology laboratory, with PCR samples in capped syringes or sterile Eppendorf containers for PCR. Posterior vitreous detachment may be induced according to local site surgeon discretion if posterior vitreous detachment is not already present. After core vitrectomy, additional laser or cryotherapy treatment may be performed if there are retinal breaks present. The choice of intraocular tamponade agent will be decided by the consultant vitreoretinal surgeon, and the choice of either air, gas, or silicone oil are options. Subconjunctival antibiotic is routinely administered in line with department guidelines, and wound sites are checked and sutured where necessary. For post-operative medication we advised that sites should prescribe their standard care medication regimen to patients following the pars plana vitrectomy.

***Standard of care-control arm***

An intravitreal antibiotic injection of two drugs: vancomycin (2.0mg/0.1 ml) and, amikacin (0.4 mg/0.1 ml), or ceftazidime (2.25mg/0.1ml) ) will be administered on the day of randomisation. According to MHRA guidance, EVIAN is not a Clinical Trial of an Investigational Medicinal Product (CTIMP) and as such routine NHS amikacin, vancomycin, ceftazidime stock may be used, without specific trial labelling. Medications must be stored in accordance with the manufacturer’s instructions and also in accordance with local policy. The safety and supply of amikacin, vancomycin, and ceftazidime will be overseen by the site’s non-trial pharmacy, but if any issues of concern arise, the Chief Investigator or Trial Manager should be informed. Intravitreal antibiotics must be administered by a qualified ophthalmologist experienced in intravitreal injections. The drug should be inspected for particulate matter and discoloration prior to injection. The injection should be undertaken in the standard manner for the investigating unit. The periocular skin, eyelid and ocular surface should be disinfected with povidone iodine 5%, following topical anaesthesia. The injection needle should be inserted 3.5–4.0 mm posterior to the limbus into the vitreous cavity, avoiding the horizontal meridian and aiming towards the centre of the globe. The injection volume of 0.05 ml should be delivered and then the needle should be held in position for at least 5 seconds to minimize reflux. A different scleral site should be used for subsequent injections.

In the Control Arm, there is the option for a participant to undergo vitrectomy surgery as part of standard of care, within 12–15 days of confirmation. The criteria for intervention would be that the vision has reached perception of light, and a surgical discussion would be carried out in conjunction with the medical and surgical retina teams.

### Trial patient information leaflet

The study has been peer reviewed by the Advancing Clinical Trials in Vision and Eyes support group at Moorfields Eye Hospital/UCL, and the ACTIVE group has reviewed the EVIAN trial patient information leaflet. Members of the public have reviewed the document, and this has been approved by the ethics committee.

### Data management

All study records should be maintained in a locked, limited-access area.

The local Investigator will act as custodian for the trial data at each site. The following guidelines will be strictly adhered to:

- Patient data will be anonymised before sharing with the central study team
- All anonymised data will be stored on a password protected computer in the clinical research facility
- All trial data will be stored and archived in line with the Medicines for Human Use (Clinical Trials) Amended Regulations 2006.

All study data will be initially entered onto either electronic or paper source documents (according to site hospital record policies). The study CRF will then be completed by an Investigator before data entry into the online Castor platform. A bespoke EVIAN trial database has been constructed on Castor. The database is a secure online database that permits entry of non-identifiable patient data pertaining to the EVIAN trial. Sites will be provided with a username and password upon registration. Users will be required to enter baseline information at week 0. Data is entered at each numbered follow-up appointment and at the final week 24 visit. Data is entered as numbers, free text, or multiple-choice selection via radio buttons or drop-down menus. It has been designed to be easy to use, safe and to facilitate compliance with good clinical practice guidelines.

All requested information must be entered on the Castor platform. If an item is not available or not applicable this fact should be indicated. Data management must comply with the UK General Data Protection Regulation. The data management team and study monitors may raise queries using the electronic system, and the study site Investigator must provide a response in a timely manner. To ensure the quality of clinical data across all participants and sites, data will be checked for consistency, omissions, and any apparent discrepancies. In addition, the data will be reviewed for adherence to the protocol. To resolve any questions arising from the clinical data, data queries and/or site notifications will be created in the database for resolution.

### Concomitant care

Concomitant medications are any prescription drugs used by a patient during the study, until conclusion of study participation (week 24) or early termination. The paper source documents and electronic case report forms will record administration of these medications. Use of concomitant treatment with a clinical device should also be recorded. Patients will be assessed for evidence of significant cataract as part of their clinical assessment. A more formal and thorough assessment for the presence of visually significant cataract should take place at month 6, allowing reasonable time for treatment and recovery before final assessment. No other experimental or investigational treatments are allowed during this study, including ocular experimental and investigational treatments in the study eye. During the study period, participants may have other non-ocular conditions manged through the GP or hospital care providers, and these will be recorded in the source files.

### Feasibility objectives

***Primary outcomes***

Our study aims to explore the feasibility and acceptability of carrying out a RCT of early surgical treatment compared to standard treatment for POE. The main aim of this feasibility study is to determine whether a future definitive trial of early vitrectomy in POE would be feasible and to determine the sample size for this definitive trial. The major areas of uncertainty in this study relates to the acceptability of patients within an emergency setting to be randomised to either of two interventions, namely intravitreal antibiotics or early vitrectomy surgery. A further area of focus will be the ability to identify, recruit and treat participants who have developed POE within the current NHS ophthalmic patient care pathway. In addition, we will collect outcome measures at six months that include visual acuity, adverse events/complications, and health economic data. The study aims to provide a signal of early PPV plus standard care intravitreal antibiotic injections compared to standard care intravitreal antibiotic injections in the management of POE.

To determine the feasibility of a definitive randomised controlled trial, the success criteria will be:

- Rates of recruitment and retention rates of participants with POE
- The willingness of surgeons to refer patients into the study at each clinical site
- The willingness of participants to be randomised to either standard of care or early vitrectomy surgery.
- To investigate the feasibility and expected effect size with which to inform the design of a definitive randomized trial of this question.

Every month, we will issue each site with a trial-specific screening log that will be tailored to our study objectives. We will capture numerical and site-specific reasons/commentary data for ineligibility based on total numbers of index POE cases each month. Sites will provide reasons for participant(s) declining to participate; and, clinician engagement.

***Secondary outcomes***

The following additional outcomes will be evaluated in the study.

- Distance Best Corrected Visual acuity
- Rate of completed follow up
- Agreement of clinicians to enrol patients who will then be randomised to 2 arms
- Rate of secondary procedures
- Rate of complications
- Health Related Quality of life (HRQoL)
- Client Services Receipt Inventory (CSRI) completion frequency and analysis

The following data will be used in a provisional exploratory analysis to identify factors (if any) predicting a beneficial outcome from vitrectomy compared to the standard care of patients undergoing intravitreal antibiotic therapy for endophthalmitis:

- Demographic data
- Type of eye surgery/procedure and/or intravitreal injection
- Baseline versus final visual acuity
- Type of culture positive causative organism

### Adverse events

All adverse events (AEs) and serious adverse events (SAEs) will be followed through to resolution or 30 days after the participant terminates from the study, whichever occurs first. The Sponsor or its designee may follow-up with the site by telephone, email, and/or a monitoring visit to obtain additional case details deemed necessary to appropriately evaluate the SAE report (e.g., hospital discharge summary, consultant report, or autopsy report). The Chief Investigator will provide an annual report of all SAEs, which will be distributed to the REC, as appropriate. Expected AE’s resulting from the Pars Plana Vitrectomy or IVAB’s will not require reporting. A list of study-specific AEs is listed below

Expected AE:

- Cataract
- Inflammation
- Retinal tear or detachment
- Intraocular pressure of ≥ 45 mmHg
- Cystoid Macular Oedema

Serious AE:

- Choroidal haemorrhage
- No perception of light vision
- Vitreous haemorrhage

AEs will be recorded in the medical records and AE form following consent until the patient has completed their final study visit. For the purpose of trial reporting, any unresolved AEs at final visit will be reviewed up until 30 days post termination. If within this timeframe the AE is resolved the date of resolution and outcome should be recorded on the AE log. After study completion, the AE records will be verified.

There will be monthly EVIAN trial management meetings to review AE/SAE reports. The adverse event profile will be monitored by the sponsor and reported to the trial steering committee on a regular interval. Patient Advisory Group meetings will be hosted by the sponsor periodically to review patient experience and AE events.

### Follow up data collection

Participants will undergo ETDRS VA testing, clinical examination, ultrasound, wide field or 7-field fundus photography, and an assessment of vitreous haze should take place in all patients at baseline, week 4 follow-up and final visit. Photography may be repeated any time during follow up visits if the examining clinician feels it to be necessary. If baseline fundus photography is not possible due to vitreous haze, then an anterior segment photograph should be taken to log the cause. Images and vitreous haze should be graded in line with the Nussenblatt grading of vitreous haze score ^12^.

### Sample size

For feasibility and pilot studies, sample sizes between 24 and 50 ^13– 15^ have been recommended to estimate a chosen parameter. We have chosen a 1:1 treatment to control ratio, therefore a total sample size of 40 would be enough to estimate the standard deviation of the outcome of at least 20 treated patients in each group, allowing for some loss to follow-up. We will also be able to estimate our expected recruitment rate (i.e. of the eligible patients how many are entered onto the trial) of 50% (95%CI: 60-80) if we approach around 80 eligible patients with acute POE.

### Statistical methods

A statistical analysis plan will be written by the trial statistician and approved prior to the analysis of any locked data. Descriptive analysis of baseline characteristics for the two arms will be reported using mean and standard deviation (SD) for approximately normally distributed continuous variable or median and interquartile range (IQR) for non-normal continuous variable (skewed). Categorical variable will be reported as count and percentage. This will be conducted to assess the adequacy of randomisation. The proportion of patients who accept the offer of randomisation will be reported with 95% confidence intervals (95% CI) computed by the exact binomial method as will all outcomes assessing feasibility. In addition to summary statistics of the secondary outcomes (similarly to the primary and baseline outcomes), all harms and withdrawals will be reported with 95% CIs. There are no planned subgroup analyses and no planned interim analysis. Patients will be analysed in the groups to which they are randomised. Missing data will be reported by treatment group with reasons for missingness described. Since this is a feasibility study, we do not plan to impute missing data.

We hope to be guided by this trial on the feasibility of scaling up the sample size to plan a full randomised control trial of the same question.

### Embedded health economic study

The health economics analysis will assess the feasibility of performing an economic evaluation of the intervention compared to current standard treatment, using accepted methods ^16^. We will analyse the cost-effectiveness of vitrectomy surgery compared to standard treatment, using a short-run time horizon (6 months, the ‘within trial’ period). Costs will be identified and assessed adopting the NHS and personal social care perspective. Costs will include NHS resource use (e.g. antibiotics, treatments, diagnostic tests, surgery, further A&E attendance, cost to treat complications and adverse events etc), collected through the trail and using hospital data and CSRI ^17^. Unit costs will be taken from standard sources. 

Main outcome measures in the trial are the visual function and the health-related quality of life (HRQL). These will be measured by administration of health-related National Eye Institute Visual Function questionnaire (VFQ-25) ^18^ and health questionnaires (EQ-5D-5L) ^19^ to assess changes in Quality Adjusted Life Years in both groups at 6-months.

Patient specific utility profiles will be constructed assuming a straight line relation between each of patients’ health-related quality of life scores at each follow-up point. The QALYs experienced from baseline to six months will be calculated as the area underneath this profile.

Cost-effectiveness will be calculated as the mean cost difference between early vitrectomy and standard care divided by the mean difference in outcomes (Visual Function/QALYs) to give the incremental cost-effectiveness ratio (ICER).

## Ethics and dissemination

Ethics approval was obtained from the West Midlands-Solihull Research Ethics Committee on November 20 ^th^ 2020 prior to trial commencement Committee (REC: 20/WM/0264) (20/11/2020), and prospectively registered (ClinicalTrials.gov: NCT04522661). A Trial Steering Committee was appointed to independently monitor progress of the trial and recommend whether there are any ethical or safety reasons why the trial should not continue. Summary results data will be included on the trial registration database within 12 months of the end of the trial. Requests for data (anonymised trial participant level data) will only be provided to external researchers who provide a methodologically sound proposal to the trial team (and who will be required to sign a data sharing access agreement with the Sponsor [Moorfields Eye Hospital]) and/or in accordance with funder guidance.

The trial results will be published in an open-access journal, in accordance with funder policy on open-access research. The trial results will be reported following the Consolidated Standards of Reporting Trials (CONSORT) guidelines. We will inform participants of the results of trial feasibility criteria. The participants will be asked if they would like to be informed of this as part of the consent process.

## Study status

Open to recruitment.

## Data Availability

No data are associated with this article.
