# Supplementary material for: A Randomised Controlled Trial of Early Vitrectomy and Intravitreal Antibiotics for Post-operative Exogenous Endophthalmitis (EVIAN study): study protocol for a feasibility trial
Source: NIHR Open Res. 2023 Sep 28;3:47. [Version 1] doi: 10.3310/nihropenres.13469.1 (PMC11320040; doi:10.3310/nihropenres.13469.1)
Supplement: Peer review history

# Reviewer report: Reviewer response for version 1

**DOI:** 10.3310/nihropenres.14613.r30711

**Published:** 2023-11-24

**Authors:** Gita Satpathy

**Affiliations:** 1 Department of Microbiology, All India Institute of Medical Sciences, New Delhi, India

This proposal is for a feasibility study, prior to a larger RCT, to assess the clinical benefits and cost effectiveness of early pars plana vitrectomy (PPV) compared to standard treatment in post operative endophthamitis (POE) occurring after any of the eye surgeries or intravitreal injections. This study is relevant because POE is the most important complication after cataract surgery, other eye surgeries and more commonly after intra-vitreal injections. Although exact prevalence of POE is not available, it is believed to be around 0.51 per thousand operations. It results in vision loss in a significant proportion of affected eyes. Therefore early intervention is essential to limit the damage.

 The seminal, “Endophthalmitis Vitrectomy Study” was conducted almost 2 decades ago in 1995 in post cataract endophthalmitis; the recommended guidelines are still being used in the standard management of POE till date. In the meantime use of intra vitreal injections for AMD and DMO (Diabetic macular oedema) has become common; these are also resulting in POE in some patients.

 In the past decade, small gauge (23,25, 27 gauge) PPV is being increasingly used along with routine use of silicon oil. These have significantly improved the outcome of vitrectomy after POE. In POES associated with surgeries for glaucoma, PPV has proven to be beneficial.

 These factors were not taken in to consideration in the 1995 EVS study. In the mean a 2005 report has shown the significant benefits of early vitrectomy (within 5 days of presentation) than later vitrectomy surgeries in POE.

 Therefore keeping in mind the advancements in vitreoretinal surgeries, there is a need for a new trial to find out the benefits of early vitrectomy in POE occurring after a larger spectrum of eye surgeries or injection procedures

Is the study design appropriate for the research question?

Yes

Is the rationale for, and objectives of, the study clearly described?

Yes

Are sufficient details of the methods provided to allow replication by others?

Yes

Are the datasets clearly presented in a useable and accessible format?

Not applicable

Reviewer Expertise:

Ocular Microbiology, Molecular Biology,

I confirm that I have read this submission and believe that I have an appropriate level of expertise to confirm that it is of an acceptable scientific standard.

# Reviewer report: Reviewer response for version 1

**DOI:** 10.3310/nihropenres.14613.r30566

**Published:** 2023-11-06

**Authors:** Hiroyuki Nakashizuka

**Affiliations:** 1 Department of Ophthalmology, School of Medicine, Nihon University, Tokyo, Japan

This study covers all ophthalmic surgeries, not just the cataract surgeries covered by the EVS; 30 years are about to pass since the EVS. During this time, vitreous surgery has advanced markedly. In Japan, vitrectomy is recommended for endophthalmitis as early as possible, regardless of preoperative visual acuity. Prospective studies of early vitrectomy such as this one are needed to evaluate the effectiveness of this strategy.

 However, there are some points that I do not understand. At the time of diagnosis of endophthalmitis, is the treatment only intravitreal injections of antibiotic even if the visual acuity is less than 35 letters? The text states that the patient is to be reevaluated after 48 hours, but the third line of the Trial Weeks section of Table 1 states 0-48 hours (from Baseline). Does this mean that vitrectomy may be performed within 48 hours? As this is a clinical trial to evaluate the usefulness of early vitrectomy, we believe that surgery should be an option earlier if the conditions are right, rather than waiting 48 hours before considering surgery. I believe that the prognosis will be better if the surgery is performed with good visual acuity, not less than 35 letters, but less than 70 letters, for example.

Is the study design appropriate for the research question?

Partly

Is the rationale for, and objectives of, the study clearly described?

Yes

Are sufficient details of the methods provided to allow replication by others?

Yes

Are the datasets clearly presented in a useable and accessible format?

Yes

Reviewer Expertise:

Vitrectomy, retinal surgery, endophthalmitis

I confirm that I have read this submission and believe that I have an appropriate level of expertise to confirm that it is of an acceptable scientific standard.

# Response

**Published:** 2023-11-06

**Authors:** MAHIUL MUQIT

**Affiliations:** Vitreoretinal Service, Moorfields Eye Hospital, London, UK

Thank you for the comments

 Re: Does this mean that vitrectomy may be performed within 48 hours?

 The patient would have tap and injection as part of standard of care on initial presentation. They would then attend at the 48hours timepoint. At this 48 hour timepoint, if they are recruited and randomised, the vitrectomy surgery would be carried out after the 48 hour timepoint as per the protocol
